# A Very Valuable Lesson for Those Who Use Anæsthetics
*Paper read before the Baltimore Academy of Medicine.


**Published:** 1888-03

**Authors:** Julian J. Chisolm

**Affiliations:** University of Maryland.


ARTICLE II.

### A VERY VALUABLE LESSON FOR THOSE WHO USE ANÆSTHETICS. *

BY PROF. JULIAN J. CHISOLM, M. D., UNIVERSITY OF MARYLAND.

R. A.——, a robust, healthy child, three years of age, was recently brought to me with a cancerous left eye. The attention of the parents was first called to the yellow appearance of the pupil eighteen months before. The gliomatous mass filled the vitreous cavity, distending the pupil, and obliterating the anterior chamber. The eye was injected and painful. The prompt removal of the eyeball was urged as the only means of protecting the child from a painful death. The operation was accepted by the parents, and the enucleation, under chloroform, accomplished after much difficulty, as the sequel will show.

The child was suffering from a bronchial trouble, but that was not deemed an obstacle to the administration of an anæsthetic. The patient was placed on the operating

* Paper read before the Baltimore Academy of Medicine.

table, his clothing loosened about the neck and chest, and chloroform was inhaled from a towel, folded in conical form, with open top. Deep sleep was soon induced.

When the anæsthesia was complete, the operation for the removal of the diseased eye was commenced. The conjunctiva was divided around the cornea, and the tendon of the external rectus muscle was being sought for, when respiration suddenly ceased. The face assumed a death-like pallor, the pulse disappearing at the same time from the wrist. Immediately the child was suspended by the feet, with body and head hanging down at an inclination of seventy degrees, while an assistant volunteered chest-compression for artificial respiration. After a few minutes signs of a feeble respiratory movement were noticed, a slight throbbing of the neck vessels was detected, and in time the child evinced its return to consciousness by crying.

He was laid on the table, but would not permit the eye to be touched without a twist of the head, evincing great irritability or sensitiveness of the conjunctiva. As the operation had to be completed, I ordered chloroform to be again administered. Chloroform narcosis was very soon re-established, but before I had time to resume the operation the child again stopped breathing and the pulse disappeared. The body, apparently, of a dead child, was once more hung up by the feet, so as to allow blood to gravitate toward the anæmic head and brain, but with no further attempts at artificial respiration. Myself and four assistants watched anxiously the pale face, to catch the first evidence of returning vitality. After some minutes I noticed that the large vessels at the root of the neck showed some fulness, then a slight thrill, and after this the first attempt at a thoracic movement appeared. In ten minutes breathing was sufficiently strong to allow the child to cry again, much to the relief of all of us.

Still, the operation which was so imperatively called for, for the future safety of the child—even the saving of its life from the ravages of cancer—was uncompleted. While the father and mother, both present in the operating room, were pleading for their child, they were made aware, by the restlessness of the patient when the eye was touched, that nothing could be done without the child going again to sleep, so I once more ordered the inhalation of chloroform. For the third time chloroform narcosis was promptly established, and was followed very soon afterward by suspended respiration and the disappearance of the pulse. Death now seemed to be complete. Immediately the child was hung up by the feet. The absolute quiet of the operating room was broken only by the lamentations of the parents. All eyes watched the face of the child. Five minutes seemed an hour, and the ashy lips showed, so far, no response. Soon after this a faint effort at respiration was observed, which became stronger with each return of the thoracic movements, and the pulse was again felt feebly at the wrist. When respiration seemed established, insensibility continuing, I had the child laid upon the operating table. As soon as the body assumed the horizontal position, the pulse, not yet strong, disappeared from the wrist, and the respiration ceased, necessitating at once a renewal of the suspension. This curious phenomenon of breathing when suspended, and becoming inanimate when the prone position was too early assumed, was repeated two or three times, respectively. For safety—for I was afraid to lay the child down—I was forced to enucleate the eye while the child was suspended with head downward, an awkward position for operating. It was some time, fully a quarter of an hour, after the operation was completed and the eye bandaged, before I could trust the child in the recumbent posture.

One of my assistants was very anxious to have whiskey injected, and had filled his hypodermic syringe for that purpose; but I declined its use, trusting to inversion alone for resuscitation. The final successful issue of this case confirmed my faith in this invaluable method, which I

had used successfully on former occasions, and hence confided in it for the protection of the patient through the trying ordeal. In all, the child must have been suspended in the inverted position for fully three-quarters of an hour. After the last suspension no further trouble ensued. The next day the child was so thoroughly himself that he left the hospital with his parents. He was brought back to the dispensary for inspection, two days afterward, a picture of health.

This case cannot be too carefully studied by surgeons who must continue to use general anæsthetics. It is one of a series occurring to me now and then—I am glad to say, at long intervals—as the consequence of chloroform inhalation.

I am a strong advocate of chloroform, believing it to be the most available remedy of this class. I recognize it as a powerful agent for evil, but at the same time I believe it to be the best of the general anæsthetics. In army life and in civil practice I have had a personal experience of at least ten thousand administrations, and without a death. For thirty years I have had charge of a surgeon hospital service, and my daily use of chloroform has been the subject of public professional observation. Sulphuric ether I have seldom used—not one hundred times in my life, and in most of these instances only to exhibit on patients the effects of the various anæsthetics in medical classes at the University of Maryland Hospital Clinic, In the last ten years I have not used it once. For painful operations of very short duration I use the bromide of ethyl, and for all others I use chloroform exclusively.

At the Presbyterian Eye and Ear Charity Hospital of Baltimore City, iu which institution I am surgeon-in-chief, I have used chloroform as often as nine times in one day. The consumption of chloroform in this hospital is computed at hundreds of pounds. A pound of sulphuric ether has never been purchased among the hospital drugs, and it is not administered in the hospital.

My rule of practice has always been to do surgical work with the least possible pain, and to refuse anæsthetics to no surgical patient. In the administration of chloroform certain rules are followed. All clothing must be loose around the neck and chest. With adults, an ounce of whiskey is given in advance. In the case of persons under thirty years of age this cardiac stimulant is omitted, unless the patient be feeble. In this hospital practice no precautions as to eating can be observed. The clinic is held at two o'clock every day. Patients are frequently sent from the dispensary to the operating room one hour after they have eaten a hearty meal. If the patient has been admitted into the hospital wards the day before operation, his dinner is withheld.

Chloroform is administered with the patient lying on his back, and as soon as narcosis is induced the pillow is taken from under his head, so that he lies in an absolutely horizontal position. Should snoring occur, indicating some difficulty in pharyngeal breathing, the chin is drawn forcibly upward. This elevation pulls the anterior wall of the pharynx, with the hyoid bone and root of tongue, forward, making for the air a clear and straight passage from the nose into the lungs. By this movement of the chin respiration becomes immediately quiet and easy. The pulling up of the chin is a much more efficient and convenient means of pulling the root of the tongue forward than by pulling out the tongue with a forceps, as is recommended by some surgeons. It is not always easy at this stage of anæsthesia to get into the mouth, as the lower jaw muscles may not be relaxed. A proper tongue forceps is not often at hand, and to tear the tongue substance with sharp-toothed and yet slipping instruments, with the soreness and swelling which subsequently follow, is an abominable practice that should be abolished. The patient's chin and your own hands are always present, and it only needs knowledge of the method to apply it, and to secure prompt and speedy relief.

The instrument used for the inhalation is a towel folded in cone form, with the apex of the cone open, so as to permit air to mingle freely with the chloroform vapor. During the administration the face is closely watched by the surgeon. If the ears remain pink, the heart and lungs must work properly; therefore, there is no need for feeling the pulse. Any failure on the part of either of these organs can be seen in the change of the complexion more quickly than it can be felt at the wrist. When the conjunctiva is no longer sensitive, the patient is considered thoroughly anæsthetized, and the administration of chloroform is stopped. In eye work the chloroform administrator must now get out of the way for the surgeon, and therefore the administration of anæsthetic cannot be injuriously continued. Herein lies one great point of safety with the ophthalmic surgeon.

As I have previously stated, I deny chloroform to no surgical patient. Prior to the discovery of cocaine as a local anæsthetic, I administered chloroform for cataract extractions, enucleations, iridectomies, squints, lid operations, passing of lachrymal probes, or, in fact, any painful operation whatever, and even for the examination of irritable eyes in children. These patients were of all ages, from infants to octogenarians, and of course represented every condition of disease and health. If restoration to sight could be obtained, operations were performed on the blind regardless of the diseased conditions of other organs. Some patients were strong, and some were very feeble, with lung, heart, and kidney diseases. No pathological lesion in any other part of the body deters me from the use of chloroform should an eye operation be required.

Cataract patients are usually old; most frequently they exhibit decided senile changes. I suppose an average of sixty years of age would represent this class of patients; seventy, seventy-five, to even eighty-five, ninety, and ninety-five, are at times the respective ages; ninety-six is the extreme age at which a successful cataract extraction has been performed under chloroform at the hospital. It is well known that fatty hearts are very frequently found in old subjects in dissecting rooms. My cataract operations now reach fifteen hundred. Of these, many must have had fatty hearts. Prior to the last two years, before cocaine came into use as a local anæsthetic, I gave chloroform in all cataract cases, and therefore must have given it to many patients with fatty hearts.

Very feeble heart pulsation, with irregularity of action, I frequently met with in old patients. With such I always increased the amount of whisky, which I administer in advance of the chloroform inhalation. I consider it much the safer practice to put whiskey into the stomach, where it is ready for use, if wanted, and where it can do no harm if not needed. I have never had occasion to inject whiskey or ether into the rectum or under the skin. The hypodermic syringe forms no part of my chloroforming apparatus.

So far, after thirty years of active surgical life, I can conscientiously say that in no single case have I had cause to regret that I chose chloroform as the anæsthetic. I always give chloroform in the presence of physicians—never alone—and most frequently with the whole surgical staff of the hospital present. Had death from chloroform occurred in my practice, it would have received, necessarily, prompt publicity. That I have never had one, and that I have never refused chloroform to any patient received into the hospital for surgical treatment, is a fact well known.

I repeat, that in my long experience I have never had a death from any anæsthetic, although I have given chloroform to over ten thousand persons of varied sanitary conditions; but I freely acknowledge that I have come very near having a fatal ending more than once. I have had four cases of sudden arrest of respiration, with failure of the heart's action, when death would have inevitably been the final result had not prompt and proper means been taken to resuscitate the patient.

Experience under these severe trials has made me a firm believer in the efficacy of inverted suspension for the restoration of life in patients apparently killed by chloroform. I feel convinced, from my own experience with this invaluable method, that many of the dead from chloroform might have been resuscitated had the surgeon hung up immediately by the feet the inanimate body, instead of wasting time in applying hypodermic injections, cold water splashings, spanking, fanning, electricity, or even attempts at artificial respiration, the remedies which text books on surgery recommend. Do any or all of these things if you will, but hang up the patient first, and that instantly, as soon as the heart and lungs fail. It is the horizontal position that is fatal in chloroform poisoning, and leads to death if the body is kept in it, as all the reports of fatal cases with chloroform show.

With myself it has become a matter of faith, and in suspension alone I now place my confidence. So far it has served me most successfully. Had I not used suspension in the four cases referred to, most of them, probably all of them, would have died. Then my percentage of fatal cases would have corresponded with the average chloroform mortality; one in twenty-five hundred cases of administration.

By suspended respiration I refer to the complete arrest of all respiratory movements. I do not mean that very feeble state of heart and lung action, accompanied by pallor of face, which frightens so many physicians, and which I know only foreshadows the approaching vomiting. This condition of depression I see with a great many chloroform patients. With me it is only a signal that a basin should be in readiness. I often hear physicians, in giving their experiences, speak of their very narrow escape from a fatal chloroform administration, meaning this stage of depression which one familiar with chloroform, from its daily administration, would almost call normal.

Many years ago I became very much impressed by certain experiments made by Dr. Nelaton—that well known French surgeon—to show that in chloroform narcosis the respiratory and cardiac centres were weakened by an anæmic condition of the nervous apparatus, the exposed brains of animals bleaching as chloroform vapor was inhaled by them to complete anæsthesia. When this whitened appearance indicated such a condition as to give but little of the blood-stimulus to the great nerve centres, their functions ceased in a regular order: First in volition, next in voluntary movement, then in general sensation, and finally in the arrest of involuntary or organic movements, including the action of the heart and lungs, and then death promptly ensued. In his experiments he found that when a number of rats had been thoroughly narcotized with chloroform, those which he would immediately hang up by the tail would slowly revive, while those left supine on the table died. If, when animation commenced to show itself in the hung up animal, the rat was laid down too soon, breathing would again cease, and the rat would die, unless immediately suspended, when the respiratory and cardiac actions would be resumed. It was only after a sufficiently long suspension, giving the brain and heart ample time to have supplied to them, by gravity, a desired amount of blood, that death could be prevented. If the animal was not already dead, suspension alone would restore animation. The knowledge of this fact is daily put into use by vivisectionists in their experiments upon animals under chloroform. The case of the child which I have reported is really in the line of these experiments, and clearly shows the danger of the horizontal position when the heart and lungs fail. The suspending of the human body by the feet to restore animation in chloroform poisoning was Nelation's great discovery, and is known as his method of restoring patients to life when, under chloroform anæsthesia, respiration has suddenly ceased. The knowledge of, and faith in, this method, has served me well on many trying occasions. To it alone I attribute my clean record of over ten thousand cases of general anæsthesia and no death.

Eighteen months since I ordered chloroform to be administered to a patient, eightly years of age, who had his right ear a mass of epithelioma. The pinna was much enlarged and an offensive, painful ulcer, with ragged outlines, covered nearly the whole surface. The object of the operation was to remove all of this fetid, discharging surface, and to close as much of the wound as possible, by quick union. His history, as given by himself, was quite a curious one of coincidences. He had been married twice. His first wife had a cancer of the breast, for which an operation had been recommended by his family physician. She died under chloroform before any incisions were made. His second wife was brought to me six years ago, suffering from a malignant disease of the socket, involving the eyeball, the eyelids, and skin of cheek. The cervical glands at angle of jaw were secondarily infected. I declined to operate, and advised against it, as no good could possibly come from it. She returned, disappointed, to her distant home. Against my advice, the local physicians urged the operation, and in her anxiety to get rid of the cancer, she yielded to their solicitations. They undertook it, and she died during the operation—they said, from the effects of chloroform. There were no blood relationship between himself and either of his two wives, and yet he also had a cancer for which an operation under chloroform had been advised. He was of a robust frame, although eighty years of age. In his desire to get rid of the fetid discharge, he submitted without hesitation to the course recommended.

First a full dose of whiskey was taken, and then chloroform was administered by the resident physician of the hospital, aided by the medical staff. I had left the operating room for a few minutes, to show to a medical visitor some case of interest in the wards, when the nurse ran to inform me that the man whom I had just left was dead. I hastened with my medical friend to the operating room. I found one of the physicians trying thoracic compression for artificial respiration on an apparently lifeless body, lying flat upon the operating table. I had this immediately stopped, and under instructions the four doctors present, with the nurse and the brother of the patient, held up the lower end of the operating table so as to incline the body and head an an angle of over forty five degrees, using at the same time all of their restraining force to keep the body from sliding off the table. Nothing else was done. With the inanimate body in the way suspended, we quietly and anxiously awaited results. In a very few minutes we had the satisfaction of seeing slight thoracic movements, then the ashy, livid face lost its death-like hue. When respiration became fully re-established, the table was lowered and the operation safely completed, no more chloroform being required in this case.

A *third case* occurred in my hospital experience eight years ago. It was that of a woman, forty-five years of age, who had suffered frightfully from repeated attacks of iridocyclitis. I had urged an iridectomy as a means of protection from suffering, but on account of timidity she had steadily refused to submit to it. After many sleepless nights of agony, and being worn out by the pain, she finally consented to be operated upon. Loss of sleep and the constant pain had enfeebled her very much. She was given two ounces of whiskey before being put on the operating table. Complete anæsthesia under chloroform was soon induced. The eye-speculum was being placed in position, when respiration suddenly ceased. No one was feeling the pulse, as I was standing over the face, watching the skin-circulation. She looked dead, and we thought her so. Fortunately, there were several physicians present, and immediately she was hung up by the feet. While I watched the effects of suspension on the face, more attempts were made, by rythmical abdominal pressure, to force air from the lungs and thereby excite a respiratory movement. This, however, was soon desisted from, being inconvenient, and, as I thought, useless. After a few minutes of suspension, respiration was gradually re-established.

The patient, brought back to life, was again laid upon the operating table. She was perfectly relaxed, and I hoped that I could do the iridectomy without any further anæsthesia. The moment I touched the eye a flirt of the head exhibited a degree of irritability, showing very plainly that it was impossible to attempt it. As the pulse by this time seemed perfectly re-established, and the stomach contained a good quantity of whiskey (there had been no vomiting), I determined again to give her chloroform. A very few whiffs from the charged towel brought on full anæsthesia, and with every promise that the various steps of the operation could now be successfully carried out. The speculum was applied, the eyeball seized, and the cataract-knife had transfixed the cornea, when respiration and cardiac action again stopped. The patient now seemed quite dead. The eye instruments were quickly removed, and the patient in an instant was hung up by the feet, with head down. No attempts were made at artificial respiration, nor were any other means used for resuscitation, but the inversion, not even throwing open the windows for fresh air. As still as death, we watched the suspended body. After a few minutes, which seemed a very long time to us, a feeble respiratory movement was detected. This slowly developed into full breathing, and brought back the pulse, and with it life to our patient.

She was again laid on the table, utterly limp, but breathing freely. When the eye was touched the head made again a sudden movement, showing a degree of conjunctival irritability which rendered the completion of the eye operation impossible. The question now before me was whether I should leave the eye with an operation half performed, or protect the patient from future suffering by completing what I had started out to do. After consultation, I concluded to perfect the operation, and, with an abiding faith in the efficacy of suspension, I ordered chloroform again to be administered. For the third time quiet sleep was quickly induced, and, fortunately, with no further complications or trouble, the operation was successfully and safely completed.

Twelve years ago, a fourth case occurred under my treatment. A gentleman brought to me his two boys, one eight, the other six years of age, both subjects for squint-operation. Such operations I frequently perform in my office, with the aid of one professional assistant. The elder boy was put to sleep under chloroform, the tenotomy of the rectus completed without trouble, and he was laid upon a lounge, vacating my reclining operating chair for the younger boy. He also bore chloroform apparently as well as his elder brother, and under its narcotic influence the squint-operation was speedily completed. After I had removed the eye-speculum, and cleansed the conjunctival sac of blood, respiration suddenly stopped, the pulse disappearing from the wrist, and accompanied by the death-like appearances which belong to this startling condition. Fortunately, I was sitting at the head of the patient, and I immediately tilted down my end of the operating chair, getting my assistant to elevate the foot-end, so as to secure an elevation of forty-five degrees, with the head of the patient downward. After a few minutes, blood gravitated into the head. By stimulating the nerve-centres, it started into action those organs so essential to life. Breathing was finally re-established, and with it the circulation.

These four cases of sudden arrest of the respiratory functions, with failure of the heart's action during chloroform-narcosis, occurring in my own individual practice, I feel assured that most of these patients would have died had they been left in the recumbent posture, regardless of what may have been done otherwise for their restoration. Fanning, fresh air, water-splashing, spanking, whiskey or ether injections, electricity, artificial respiration—all of them the remedies which physicians rely upon—go for very little provided the patient be left supine. General experience, unfortunately, has too often shown this. In my experience in chloroform, in cases of suspended animation, all of these means for resuscitation are useless, provided the patient be hung up by the feet without any loss of time, so that blood may flow to the anæmic head and heart and stimulate the nerve-centres before the vital spark goes altogether out. A fire cannot be rekindled by adding fuel if there be no live coals in the grate. Fortunately, suspension of the body needs no preparation nor apparatus for its immediate application. It only needs vigilance on the part of the operator. Should fright make him forget his duty, then precious minutes are lost in trying useless remedies, and these precious minutes can never be recalled.

That all of my cases of apparent death from chloroform should have recovered, is not merely good luck, nor is it accidental. I know that chloroform, ether and ethyl are powerful agents for good, and also for evil. I am sure that I can kill any patient by the abusive or careless administration of either of these remedial agents, just as I am sure that I can be burned by any kind of heating apparatus which I am so dependent upon for genial warmth in winter.

The successful administration of an anæsthetic does not consist merely in holding before the nose of the patient a cloth with the narcotizing agent poured upon it. Skill, care, prudence, judgment and courage in time of need, are all necessary to guard the narcotized patient from danger. Too little of the anæsthetic—not enough to protect the important vital centres from the influence of painful reflex actions—is as dangerous as an overdose of the narcotic inhalant. Many of the fatal accidents occur in the hands of timid physicians or dentists who are afraid to administer enough of the anæsthetie to secure the stage of safety, the immunity from reflex disturbances, and who lose their heads in fright when the danger which their want of confidence has induced presents itself.

The lesson which I would impress upon every one who uses chloroform, sulphuric ether, or the bromide of ethyl for general anæsthetic purposes is, that prompt suspension, with head down, is the remedy for suspended animation suddenly coming on during acquired narcosis.

No surgeon recognizing the responsibility of his work should ever give an anæsthetic without having some one present. Should there be any sudden and alarming weakening of the heart's action and of respiration—for they always go together—without a minute's delay hang up the patient. Should the patient be bulky, and should there not be force enough present to elevate the foot of the table or bed, be the patient man or woman—while you stoop, throw their legs over your shoulders, hang on to their feet in front of you, and then lift yourself. The patient's body, as you get on your own feet, will hang from your back, with the head down. Now you have time to call for more help, if you need it, Never wait for the help to come before you practice suspension, because with the moment's delay your patient may have passed from dying into death, from which there will be no more earthly awakening. When too long delayed—and one minute is a fatal loss of time—suspension is as useless as the other recommended remedies, and can then do no good.

Should the case have been one of needless fright, with only weakening, and not suspension, of the vital functions, no harm has been done. The feeble pulse will always respond promptly to the suspension. It is my constant practice to use suspension for restoring strength to the heart's action after the administration of chloroform, where there is cardiac depression and weak breathing. I use this means of restoring vigor where others use the more objectionable and less efficient hypodermics of whiskey or ether, or the inhalations of nitrate of amyl. It is very instructive to observe how promptly the pallor leaves the face, and how strong the pulse will become, as blood gravitates toward the head. Should vomiting occur when the head is hanging down, this suspended position is better for the patient than when lying upon the table, because there is no fear of food-particles getting into the larynx. Inversion of the body gives the contents of the stomach free vent.

Such confidence do I feel in the value of suspension with chloroformed subjects that I am sometimes disposed to believe that the vital centres cannot fail with the head hanging down.

Not long ago, in the presence of the medical class of the University of Maryland, I removed by ligature a very large staphyloma from a child one year old. It was the result of purulent ophthalmia of the newly born. The prominence of the opaque cornea was so great that the lids could hardly close over it. The summit of the tumor was being irritated by the constant friction of the lids in winking, and its removal became necessary for the comfort of the child. Under chloroform-anæsthesia I transfixed the eye-ball at the base of the tumor by two long, curved needles, placed at right angles to each other. Besides these, acting as a shoulder, I applied the ligature for the strangulation of the tumor. The medical class could not see from the benches the various steps of the operation upon so small a portion of the body and from the large number of students present only a few could have crowded around the operating table. After the ligature had been secured, and before the needles were withdrawn, I did not hesitate to hang up the infant by the heels, with head suspended vertically downward, and then walked with it in front of the benches, so that the students could inspect the eye. To the uninitiated this would appear a heartless and dangerous proceeding. My experience and consequent faith in suspension had taught me that this inversion was the safest position for the narcotized child during the tedious inspection.—*Medical Record.*

